# Proton beam therapy and dentofacial development in paediatric cancer patients: A scoping review

**DOI:** 10.1016/j.ijpt.2024.100107

**Published:** 2024-06-06

**Authors:** Emma Foster-Thomas, Marianne Aznar, Bernadette Brennan, Lucy O’Malley

**Affiliations:** 1 NIHR Doctoral Fellow in Restorative Dentistry, Manchester University NHS Foundation Trust, UK; 2 Adaptive Radiotherapy, University of Manchester, Division of Clinical Cancer Science, School of Medical Sciences, UK; 3 Consultant Paediatric Oncologist, Royal Manchester Children’s Hospital, UK; 4 Health Services Research, School of Medical Sciences, The University of Manchester, UK

**Keywords:** Dentofacial development, Toxicity, Paediatric, Head and neck cancer, Proton beam therapy

## Abstract

**Purpose:**

It is known that radiation to dentofacial structures during childhood can lead to developmental disturbances. However, this appears to be a relatively subordinated research subject. For this reason, this review aims to establish the current evidence base on the effect of PBT on dentofacial development in paediatric patients treated for cancer in the head and neck region.

**Materials and methods:**

A comprehensive search was undertaken to identify both published and unpublished studies or reports. A single reviewer completed initial screening of abstracts; 2 independent reviewers completed secondary screening and data extraction. A narrative synthesis was then conducted.

**Results:**

82 records were screened in total, resulting in 11 included articles. These articles varied in terms of study design and reporting quality. Owing to both poor study reporting and limited patient numbers, it is not possible to determine the effect of cancer diagnosis, chronological age at treatment, radiation dose or treatment modality on the incidence of facial deformation or dental development anomalies.

**Conclusion:**

Disturbances in dentofacial development are an under-reported toxicity in paediatric cancer survivors treated with PBT to the head and neck. There is a need for more research on dentofacial toxicity reporting, focused on the impact of treatment age, radiation dose, concurrent therapies, and the subsequent impact on quality of life.

## Introduction

### Rationale

When planning any oncology treatment in patients with a good prognosis, attention should be given to minimising late adverse effects (AEs). It is understood that younger children are more sensitive to radiation therapy due to the vulnerability of their growing tissues. With advances in paediatric cancer treatments and subsequent improved outcomes, more patients are potentially living with late AEs.^1^ For this reason, childhood cancer survivors require lifelong monitoring to limit the consequences of the late AEs of their tumour and/or treatment and improve quality of life (QoL).^2^

Radiotherapy can cause growth disturbances to facial bones and soft tissues of the face, resulting in facial deformation/asymmetry.^3^ Asymmetrical growth of the face is often exacerbated by hypoplasia of the soft tissues. Facial deformation can be a very debilitating late AE for survivors, which is often complicated by the limited available reconstructive options.

Dental development anomalies can also occur as a sequalae to radiotherapy to the HN region, however this is a relatively subordinated subject in research.^4^ It is thought that the extent to which dental problems arise depends on the chemotherapy protocol, the radiotherapy dose, and the developmental stage of teeth during oncologic therapy.^5^ However, relatively little is known about the frequency of occurrence and the impact of treatment on dentofacial development. This not only makes it challenging for clinicians to fully inform patients of potential toxicities, but may present challenges from a service planning point of view.

It is important that medical and dental professionals involved in the management of paediatric cancer survivors are aware of the dental and facial growth consequences of radiotherapy and the age dependency of the specific regional effects. Considering the physical properties of proton beam therapy (PBT) and the planned reduction of dose to adjacent normal tissues,^6^, ^7^, ^8^ the incidence and potential severity of dentofacial toxicities following PBT may differ to that in conventional radiotherapy (XRT). It is therefore important to establish the current evidence base on the prevalence of dentofacial AEs from PBT to the HN region.

### Objectives

The main research question that this scoping review addresses is ‘What is the current evidence on the effect of PBT on dentofacial development in paediatric patients treated for cancer in the HN region?’. The following sub-questions are also addressed:

1. How are dentofacial anomalies identified and classified?
2. How does the cancer diagnosis affect dentofacial development?
3. How does age at the time of PBT affect dentofacial development?
4. Is there any evidence to suggest that treatment modality affects dentofacial development (single / multimodality)?
5. What QoL measures are reported for patients with dentofacial anomalies?

## Methods

As the review question is broad, a scoping review approach was deemed more appropriate to identify the types of evidence available and to examine how research on this topic has been conducted.^9^ An a priori protocol was developed and agreed upon by all authors, but this was not published.

To be included, a study needed to report upon dentofacial development disturbances / anomalies in paediatric cancer survivors aged 15 years and below at the time of diagnosis of cancer in the head and neck region (including head and neck, brain and central nervous system, eye and musculoskeletal cancers) treated with PBT alone or in combination with non-PBT radiotherapy and/or radiation-free therapy (surgery, chemotherapy, immunotherapy). To identify all relevant literature on disturbances in dentofacial development, all types of study design meeting the eligibility criteria were considered for inclusion. No restrictions on publication language or date were placed. As it is not yet known how long, if at all, it takes for anomalies in dentofacial development to present, no minimal follow-up time was required for study inclusion.

The primary outcome measure was the presence of dentofacial development anomalies including facial deformation (soft tissue and bone hypoplasia), abnormal tooth eruption pattern, tooth agenesis (hypodontia), disturbance in crown development (shape, size, enamel hypoplasia) or disturbance in root formation (agenesis, stunting, incomplete apical development). In addition to the primary outcome, the following outcomes were also included: investigations used, the effect of age at time of PBT, the effect of cancer diagnosis, the effects of different treatment modalities (i.e., PBT alone / PBT in combination with other therapies / PBT for reoccurrence) and reported QoL measures.

A comprehensive search was undertaken by two authors (E.FT and L.O) in February 2021 (and re-run in December 2022) to identify both published and unpublished studies or reports of dentofacial development anomalies following PBT to the HN region. The search strategy was formulated with the support of a librarian at The University of Manchester and tailored for searches on the following electronic databases: MEDLINE Ovid, Embase Ovid, Cochrane Central Register of Controlled Trials, National Cancer Institute, Scopus/Science Direct and Google Scholar. The search strategy for MEDLINE Ovid can be found in the supplementary material (Table 1). Unpublished data on clinical trials were sought via searches of the US National Institutes of Health trials register (ClinicalTrials.gov) and the WHO International Clinical Trials Registry Platform. Relevant conference proceedings were searched via Embase and the Web of Science, and abstracts of dissertations and theses were searched via the ProQuest database. Additionally, British Library and Open Grey were searched. Hand searching of the reference lists of identified records was also undertaken to identify further studies that may have been missed from electronic searches.

### Assessment of relevance

Following completion of searches, the identified search result citations were uploaded to Endnote X9 Software to allow identification and removal of duplicate searches. Initially, the titles and abstracts of the identified results were screened by a single reviewer (E.FT) and obviously irrelevant titles were removed. Following this, a second round of screening was completed independently (E.FT and L.O) and any disagreements were resolved through discussion. Full texts were sourced for all records marked as ‘unclear’ and to ‘include’.

Double data extraction (E.FT and L.O) was completed through structured data extraction forms developed by the authors. The following data was extracted:

• Study characteristics including publication date, country of origin, aim, study type, sample size, presence of control and method of detecting dentofacial anomalies.
• Participant characteristics including sociodemographic information, primary oncological condition, treatment/s received, total radiation dose, treatment duration, follow-up duration, details of dentofacial anomalies and any QoL assessments.

### Synthesis of results

As this review aimed to determine the size and severity of the potential problem, data on numbers was collected. In addition to this, a narrative synthesis pertaining to the remaining outcome measures has been reported.

## Results

**Fig. 1:**
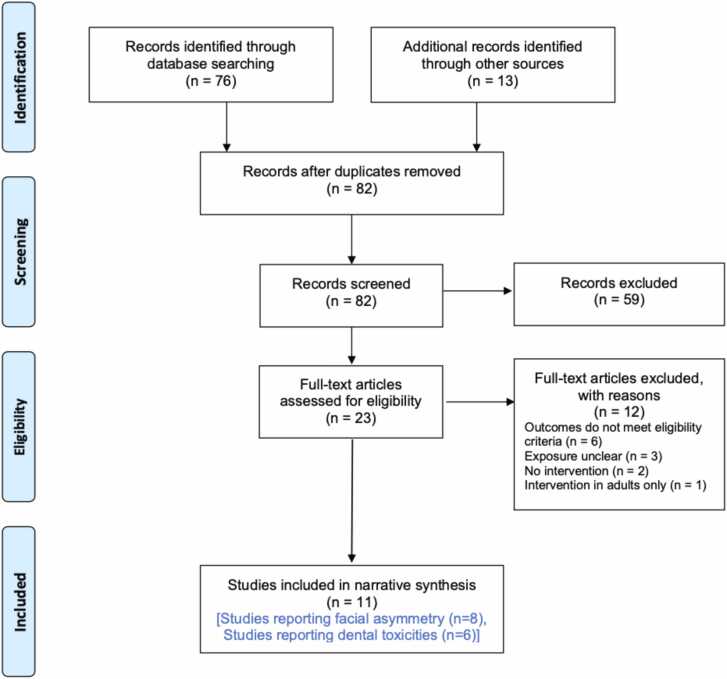
PRISMA flow diagram^10^ for the scoping review outlining the databases searched, the number of abstracts screened, the full texts retrieved and the final number of included studies.

The PRISMA flow diagram presented in Fig. 1 outlines the results of the four-phases.^10^ Following removal of duplicates, 82 records were initially identified. Initial screening led to the exclusion of 59 records which either did not include or did not clearly state whether participants treated with PBT were included. This left 23 records for double screening. A further 12 records were then excluded, leaving 11 records that met the inclusion criteria.

### Characteristics of included studies

A summary of the characteristics of the included studies is provided in Table 1 (n = 11). Of the 11 studies, 6 were conducted in treatment centres in the United States of America,^11^, ^12^, ^13^, ^14^, ^15^, ^16^ 2 in Japan,^17^, ^18^ 2 in Switzerland^19^, ^20^ and 1 in the Netherlands.^21^ However, as many of the included patients were referred for PBT from both out of state and overseas, it is not possible to conclude the patient’s geographical origins. In terms of study design, the most prominent study design found was prognostic in nature. For this scoping review, a prognostic study was defined as an observational non-randomised study that examined selected variables or risk factors in a specific cohort and assessed their influence on clinical outcomes. A summary of the characteristics of included studies can be found in the supplementary material.

**Table 1:** Summary of the characteristics of the included studies (n = 11).

Citation	Location of study	Treatment centre/s	Treatment period	Study design	Focus of study on adverse effects of intervention	Sample size	Presence of control	Follow up period	Frequency of follow-up	Method of detecting dentofacial anomalies
Childs 2012	Boston, USA	Massachusetts General Hospital	1996 - 2005	Prognostic	Yes	17	No	5 years (range: 2 – 10.8 years)	6,12,18, 24, 36, 48, 60 months	Medical notes and physician questionnaires
Fitzek 2000	Boston, USA	Harvard Cyclotron Laboratory	Nov 1986 – Dec 1991	Non-randomised trial	Yes	6	Yes	Median 9 years (range: 6.6 to 11 years)	Unknown	Serial facial photographs
Fukushima 2017	Tsukuba, Japan	University of Tsukuba Hospital	1983 - 2011	Prognostic	No	32	No	Median 51 months (range 0-340)	Unknown	Unknown
Hol 2020	Amsterdam and USA	University of Florida Health Proton Therapy Institute	2007 - 2018	Prognostic	Yes	17	No	Mean 2.9 years (range 0.8 – 3.2)	Unknown	MRI
Hoogeveen 2020	Amsterdam, The Netherlands	Academic Medical Center Amsterdam	Cases treated between 1995 - 2020	Case series	Yes	1 (5 cases in series)	N/A	12.5 years	1, 5.3 and 12.5 years	Panoramic radiographs
Kharod 2019	Jacksonville, USA	University of Florida Health Proton Therapy Institute	2008 - 2018	Prognostic	Yes	25	No	Mean 3.7 years (range 0.26-8.3)	Unknown	MRI
Leiser 2016	Switzerland	Paul Scherrer Institute	Jan 2000 – Dec 2014	Prognostic	Yes	83	No	Median 55.5 months (range 0.9-126.3)	Unknown	Unknown
Ludmir 2019	Houston, USA	MD Anderson Cancer Center	2006 - 2015	Prognostic	No	46	No	Median 3.9 years (range 0.5-8.9)	Unknown	Unknown
Mouw 2017	Boston, USA	Massachusetts General Hospital	1986 - 2021	Prognostic	No	12	No	Mean 12.9 years (range 4.8-22.2)	Unknown	MRI volumetrics and external cephalometrics
Oshiro 2011	Tsukuba, Japan	University of Tsukuba Hospital	2003 and 2005	Case series	No	2	N/A	(1) 4.5 years (2) 6 years	Unknown	Panoramic radiographs
Thompson 2013	Switzerland	Paul Scherrer Institute	2003 and 2009	Retrospective	Yes	14	N/A	Median 5 years (17-90 months)	Unknown	Individual contouring of teeth on CT slices

### How are dentofacial anomalies identified and classified?

For the 6 studies that discussed dental development anomalies, only three specified how those anomalies were observed. Only one study reviewed sequential panoramic radiographs.^21^ From the radiographs of forty-two survivors of paediatric HN rhabdomyosarcoma (RMS), the authors developed an inventory of manifestations of disturbed dental development following chemotherapy and radiotherapy. This inventory classified the disturbances as moderate or severe. Although this inventory will allow a degree of subjectivity when determining severity, it does provide useful, albeit limited examples of dental anomalies relating to both crown and root development.

Thompson et al.^20^ was the only study to utilise pre-treatment and follow-up computed tomographic (CT) imaging to assess dental toxicity. The authors manually contoured individual teeth using axial CT slices (2 mm thickness) for fourteen paediatric patients aged 1 to 16 years who received pencil beam scanning PBT. All teeth, except the third molars, were identifiable using this approach in patients aged ≥ 3 years. For the ten patients that had visited a dentist during the five-year follow-up period, two had abnormal or missing teeth and one had asymmetrical delayed tooth eruption.

Childs et al.^11^ was the only study to review clinical notes to identify AEs. In addition to reviewing the follow-up clinical notes from the referring physicians of parameningeal RMS patients, the authors also asked a series of questions, reviewed laboratory results and images to identify the late AEs in ten patients. However, the authors do not specify the investigations from which the following dentofacial anomalies were identified: mild facial hypoplasia (*n = 7*) and failure of permanent tooth eruption adjacent to the treatment field (*n = 3*). Additionally, these disturbances are not further detailed. For example, the site of tooth eruption failure, the number of teeth affected and dosimetric data to these sites.

**Fig. 2:**
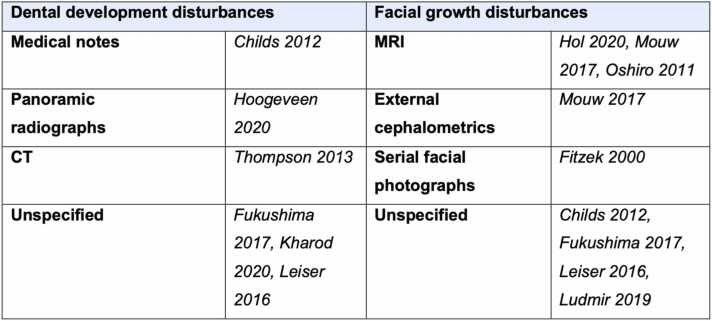
Methods of detecting dentofacial anomalies.

Facial growth disturbances are discussed in 8 studies.^11^, ^12^, ^13^, ^15^, ^16^, ^17^, ^18^, ^19^ Review of follow-up magnetic resonance imaging (MRI) was the most commonly adopted approach for detecting facial growth disturbances, being used in 3 studies^13^, ^16^, ^18^ (Fig. 2). As well as utilising MRI orbital volumetrics for the twelve patients treated for retinoblastoma, Mouw^16^ further investigated the effects of treatment on facial deformation via cephalometric analysis.

**Fig. 3:**
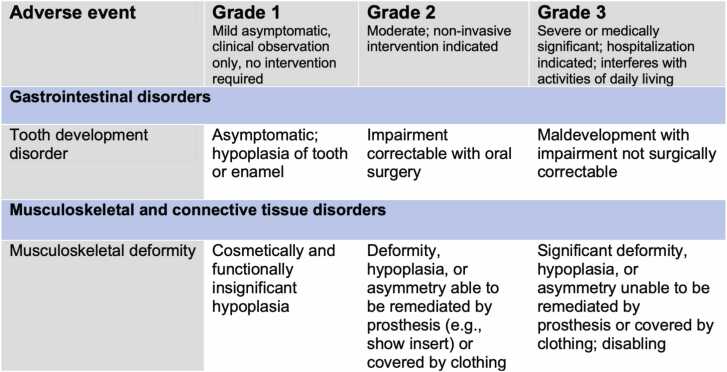
Reproduced CTCAE grades (severity of adverse effects) for tooth development disorder and musculoskeletal deformity (Version 5.0).

The investigations used to detect the reported dentofacial anomalies were often not described in the included studies. For example, in 3 of the studies^14^, ^17^, ^19^ reporting dental development anomalies, the methods of detecting these is unknown. Additionally, 50 % of the studies reporting facial growth disturbances do not describe how they were identified.^11^, ^15^, ^17^, ^19^ Only one study^17^ used the Common Terminology Criteria for Adverse Events (CTCAE) (version 4.0) to grade the identified reported facial and dental toxicitises. Fig. 3 outlines the CTCAE descriptions assigned for grading tooth development disorders and musculoskeletal deformities (i.e. facial deformation).^22^

### Does cancer diagnosis have an impact on dentofacial development?

From the included studies, it is not possible to determine if cancer diagnosis influences the incidence of dentofacial anomalies. Despite reporting the incidence of dentofacial anomalies diagnosed in the cohorts, not all authors specify the cancer diagnosis and site when describing the anomaly. Table 2 in the supplementary material outlines the cancer diagnoses reported in the studies as well as the description of the dentofacial anomalies. For example, Fukushima et al.^17^ assessed the comorbidity of thirty-two childhood cancer survivors treated with PBT to the brain, head, or neck. Of these survivors, facial deformity was reported in 25 % (*n = 8*) of patients. It can be determined that two of the patients with facial deformity (grade 1 and grade 3) had Ewing sarcoma and one patient (grade 3) had RMS. Facial deformation was also reported in children treated for retinoblastoma.^12^, ^16^

It could be speculated that there is a relatively high risk of facial deformation in those patients treated with PBT for head, neck, or brain RMS. Childs et al.^11^ identified facial hypoplasia in 41 % (7/17) of patients diagnosed with parameningeal RMS. Leiser et al.^19^ also reported facial hypoplasia in 19.5 % (9/46) of patients diagnosed with parameningeal RMS and 29.4 % (5/17) of patients with orbital RMS. Hol et al.^13^ also found that despite the planned dosimetric advantages of PBT, orbital asymmetry still developed in many survivors of embryonal RMS, with orbital volume differences on the irradiated side in 94 % (16/17) of patients. Hoogeveen et al.^21^ and Ludmir et al.^15^ also describe bone hypoplasia and facial deformation in patients with RMS, however, do not specify the subtype of RMS.

### How does chronological age at the time of PBT affect dentofacial development?

The study of fourteen patients by Thompson et al.^20^ found that the most severe dental abnormalities (tooth aplasia and delayed eruption) were found in those patients who received the highest doses at the youngest ages. From this limited data, they suggest a tolerance dose threshold of 20 to 30 Gy to the tooth (mean dose, RBE=1.1) in children aged < 4 years. Interestingly, no dental abnormalities were detected in children aged ≥ 4 years despite having received doses over 48 Gy(RBE=1.1).

Hoogeveen et al.^21^ presented a case which supports the relationship between chronological age and radiation dose. On review of a patient diagnosed with a spindle cell RMS in the right nasopharynx at aged 3.8 years and treated with only PBT up to 50 Gy(RBE=1.1), there were multiple dental development disturbances. From the panoramic radiographs taken 5.3 years and 12.5 years after treatment completion, it is evident that the roots of multiple teeth did not develop in length. This case highlights that teeth in an earlier development phase at the time of PBT are more severely compromised, with some failing to erupt.

Oshiro et al.^18^ report tooth atrophy in the right posterior mandible 6 years following PBT in a 13-year-old patient treated for nasopharyngeal carcinoma. From the dosimetric data provided, the right mandible received approximately 40 Gy(RBE=1.1). It is possible that the development of the unerupted lower right third molar has been stunted, however the radiographic appearance of the lower right second molar is consistent with dental caries not a developmental disturbance. The exact age of the four patients identified with dental toxicities by Fukushima et al.^17^ and Kharod et al.^23^ is not reported. Only the median age at time of PBT is reported, 5.3 years (range 0.7 - 14.6) and 5.9 years (1 – 21.7), respectively. Owing to the large range of ages in these studies, it is not possible to determine if chronological age influenced the patient’s susceptibility to dental toxicity.

Not only is the age at the time of radiation not provided in many of the studies, the exact age of when facial growth disturbances were observed is also poorly reported.^11^, ^12^, ^15^, ^17^, ^19^ The median ages of all the patients at diagnosis and presumably treatment with reported facial disturbances at follow-up was < 16 years. In a cohort of seventeen RMS patients, findings suggest that orbital asymmetry, defined by differences in orbital volume, will still develop in many survivors of embryonal RMS who receive > 40 Gy(RBE=1.1) to the orbital rim.^13^ The findings of this study are limited in that the patients were approximately 9 years old at the time of follow-up and therefore had not yet gone through their pubertal growth spurt, meaning further growth of the orbit is expected. Interestingly though, it was found that orbital volume differences did not correlate with age at treatment.

### Is there any evidence to suggest that treatment modality affects dentofacial development?

Of the included studies, 9 reported the outcomes following multimodality treatment.^11^, ^13^, ^14^, ^15^, ^16^, ^17^, ^18^, ^19^, ^20^ In the studies reporting dental anomalies, there was no discussion about potential confounding factors, specifically inductive or concurrent chemotherapy. The influence of chemotherapy is important considering that dental development disturbances have been shown to be associated with chemotherapeutic drugs.^24^ From the reported information in the aforementioned 9 studies, it is not possible to determine if the disturbances occurred due to the PBT alone, the chemotherapy or both treatments. Interestingly though, in the case series by Hoogeveen et al.,^21^ localised dental disturbances were also reported in patients treated with intensity-modulated photon therapy and chemotherapy in the regions that received the higher radiation doses. It therefore may be the case that the chemotherapy schedules in these patients did not contribute to the dental sequelae since more generalised dental effects would be anticipated. However, without accurate dosimetry at the individual tooth level it is not possible to determine the radiation dose (if any) to these sites. Furthermore, the effects of other agents are outside of the scope of this review.

As with dental anomalies, there was no discussion about potential confounding influences in those studies reporting facial hypoplasia and/or asymmetry. Mouw et al.^16^ did not find a systematic pattern of asymmetry in any of the recorded metrics based on treatment modality, enucleation, both or neither. Some authors acknowledged the challenges of determining the differences in toxicity rates with PBT and XRT, especially for rarer disease types like Ewing Sarcoma where there is very limited published data on treatment-related morbidities.^23^

### Are any QOL measures reported for patients with dentofacial anomalies?

QoL was assessed and discussed in 3 of the included studies.^16^, ^17^, ^19^ Versions of the PedsQL™ Pediatric Quality of Life Inventory were used, specifically the generic core and cancer modules.^25^ The cancer module includes three questions on ‘perceived physical appearance’. Although no significant difference between the child- or parent proxy-reported outcomes and those of a healthy cohort was observed in these studies, lower ‘body image’ scores were reported in the children treated for RMS in the study by Leiser et al.^19^.

The QoL scores published by Fukushima et al.^17^ suggest that facial deformity may not affect QoL since the scores were not correlated with the patient’s current comorbidities. However, it is not possible to correlate the published QoL scores to the patients with reported dentofacial anomalies. Furthermore, although the PedsQL allows a non-specific assessment of facial appearance, it is not specific for measuring health-related QoL in individuals with dentofacial anomalies.

### Quality of included studies

**Fig. 4:**
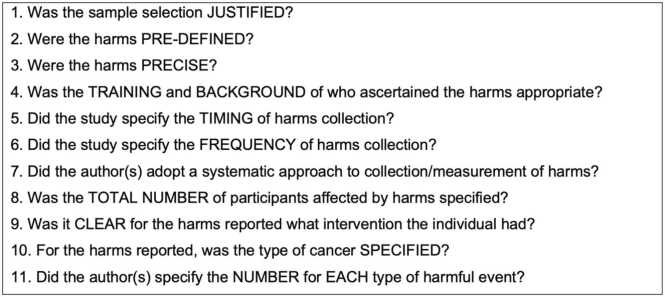
Developed quality assessment tool for scoping review.

Due to the variation of sources included and the challenges in classifying these sources in terms of study design, a quality assessment tool was developed by two authors (E.FT and L.O). As the focus of this scoping review was on AEs, the developed quality assessment tool was adapted from the McMaster tool for assessing quality of harms assessment and study reporting.^26^ The 11 questions considered in the quality assessment are outlined in Figure 4.

A summary of the quality assessment for the 11 included studies can be found in Table 2. All studies met question 8,9 and 11. The biggest area of uncertainty related to the appropriateness of the training and background of the individuals who identified the harms in the studies, with this only being outlined in 3 studies.^13^, ^16^, ^18^ Without experience of assessing panoramic radiographs for example, there is the risk that dental disturbances are missed or incorrectly classified.

**Table 2:** Summary of quality assessment outcomes for each included source.

**Citation**	**Q1**	**Q2**	**Q3**	**Q4**	**Q5**	**Q6**	**Q7**	**Q8**	**Q9**	**Q10**	**Q11**
**Childs 2012**	Unclear	No	No	Unclear	Unclear	Unclear	Unclear	Yes	Yes	Yes	Yes
**Fitzek 2000**	No	No	No	Unclear	No	No	No	Yes	Yes	Yes	Yes
**Fukushima 2017**	Unclear	Yes	No	Unclear	Yes	No	Unclear	Yes	Yes	No	Yes
**Hol 2020**	Yes	Yes	Yes	Yes	No	No	Yes	Yes	Yes	Yes	Yes
**Hoogeveen 2020**	Yes	No	Yes	Unclear	Yes	Yes	Yes	Yes	Yes	Yes	Yes
**Kharod 2019**	Yes	Yes	No	Unclear	No	No	Unclear	Yes	Yes	Yes	Yes
**Leiser 2016**	Yes	Yes	No	Unclear	No	No	No	Yes	Yes	Yes	Yes
**Ludmir 2019**	Yes	Yes	No	Unclear	No	No	Unclear	Yes	Yes	Yes	Yes
**Mouw 2017**	Yes	No	Yes	Yes	Yes	No	Yes	Yes	Yes	Yes	Yes
**Oshiro 2011**	Yes	No	No	Yes	Yes	No	No	Yes	Yes	Yes	Yes
**Thompson 2013**	Yes	No	No	Unclear	Yes	No	Yes	Yes	Yes	Unclear	Yes

The harms reported were only precisely outlined in 3 studies^13^, ^18^, ^21^ with other studies referring to more non-specific harms, for example “surgical dental procedures”. An area requiring improvement in all studies but one (^21^) is in the reporting of the frequency of harms collection. Without reporting the timings and frequency of harms collection, it is not possible to determine if there is a critical point after PBT in children of certain ages in which dentofacial anomalies may be identified and if there should be a minimal follow-up period.

## Discussion

From our knowledge, this is the first review focused on the impact of PBT on dentofacial development in paediatric patients treated for cancer in the HN region. It is therefore useful for medical and dental professionals involved in the care of these patients in providing a summary of current understandings of late dentofacial AEs.

There is no agreed approach for the identification and classification of dentofacial anomalies. Although one study was unique in developing a radiographic inventory of manifestations of disturbed dental development identified on panoramic radiographs in the reviewed cohort, it does not include all possible dental anomalies that can occur. More recently, it has been recommended that the incidence of each type of dental anomaly should be reported separately.^4^ In addition to this, no consistent approach for identifying and classifying facial growth disturbances was identified in this review. Interestingly, none of the included studies utilised 3D stereophotogrammetry (3D photography) to quantify facial deformation. Considering its ease of use, this modality may offer a solution for quantification of facial deformation via the use of dense surface models.^27^To address this unmet need, the development of a standardised, efficient approach for the reporting of dentofacial anomalies clinically and radiographically would allow for comparison of dental AEs across radiotherapy centres.

The CTCAE which is frequently used at follow-up appointments in the UK for reporting of AEs, relies on accurate interpretation of the grading definitions, a thorough reporting process in terms of the questions asked to patients and patient response.^28^ However, from reviewing the definitions reproduced in Figure 3, it can be challenging for a clinician to use the CTCAE criteria alone to grade facial deformation, since the definitions relate to management with a prosthesis only, and do not refer to facial reconstruction options (for example, fat transfer). With only one study using the CTCAE to report dentofacial toxicities and there being no consistent approach in the classification of toxicities, comparing findings between the studies is challenging.

Radiation toxicity to the teeth appears to vary with age and dose. This relationship though is complex since dental and chronological ages may differ and teeth may exist in a range of developmental phases at the time of treatment. Therefore, the teeth may have different susceptabilities to toxicity.^20^ Consequently, establishing predictive dose-toxicity modelling may be more complex as specific teeth could have different susceptibilities in patients of the same age. Not only is the age at the time of radiation not provided in many of the studies, the exact age of those patients with dentofacial anomalies is also often poorly reported. Therefore, more evidence is needed to conclusively determine how chronological age at the time of PBT affects dental development. Furthermore, it is important to appreciate the complexity of dental development. Any modifications in the genetic signalling pathways during tooth development can lead to dental anomalies.^29^ These modifications may be a result of cancer treatment or another childhood illness. To improve one’s confidence that a dental anomaly has arisen as a result of cancer therapy, a detailed patient history and review of pre-treatment dental radiographs is required.

It is known that radiotherapy to the teeth increases the risk of dental anomalies in a dose-dependent pattern. Exposures to radiation doses ≥20 Gy in survivors < 10 years at diagnosis led to a significantly increased risk of developing 1 or more dental abnormalities, with an odds ratio (OR) of 5.6 (95 % CI, 3.7 - 8.5) in those aged ≤5 years and an OR 9.6 (95 % CI, 4.1 - 22.4) in those aged 6 to 10 years.^30^ However, these findings from the Childhood Cancer Survivor Study (CCSS) on patients treated from 1970 to 1986 do not include treatment with PBT. Although the findings by Thompson et al.^20^ are limited to only fourteen paediatric patients, it does support the dose-dependent pattern and increased risk of dental abnormalities in younger patients found in the CCSS. To be able to propose dose constraints for the facial bones and teeth, more detailed dose-effect analysis is required.

The management protocols of many paediatric HN cancers consist of multimodality treatment. It has previously been reported that XRT and chemotherapy are independent risk factors for adverse oral and dental sequalae among childhood cancer survivors. An interactive effect between XRT to the teeth and chemotherapy has only been found in the outcomes microdontia and enamel hypoplasia.^31^ This review was not able to conclude how treatment modality affects incidence of dentofacial anomalies, highlighting the need for further investigation. This finding is supported in the review by Milgrom^4^ which recommends further investigation into the assessment of risk of dental toxicity associated with various chemotherapeutic agents, given concomitantly and sequentially after radiotherapy. It is the recommendation of the authors that this further investigation reviews the prevalance of dental anomalies in paediatric cancer patients treated with chemotherapy, with a focus on age at treatment, follow-up duration and chemotherapy regimen.

Despite 8 studies reporting facial deformation in patients treated with PBT, it was the focus of only one study. This study focused on orbital volume and aimed to characterise the dose-effect to the bones responsible for periorbital appearance.^13^ Owing to the poor reporting of late effects in most of these studies, it is not possible to determine the site or severity of the reported facial asymmetries without clinical photographs, stereophotogrammetric image or scans. Furthermore, only one study,^17^ albeit with limited detail, stated that surgery was undertaken in one patient as a direct result of the ‘facial deformity’.

There may potentially be an increased concern of facial deformation with PBT compared to XRT due to the absence of exit dose. This differential radiation dose to ipsilateral and contralateral bony structures may result in greater facial deformation.^11^ The cost of preserving organ function and increased risk of worsened cosmetic results has previously been raised.^32^ Both this review and PENTEC suggest that further study is very important to understand the impact of RT dose on facial deformation, as well as other toxicities experienced by survivors of head and neck PBT and XRT.

QoL was only assessed in 3 of the identifued studies with the use of the PedsQL™ to non-specifically assess the toxicities. This review highlights the need for an outcome reporting tool specific for dentofacial toxicities, which could be used by both oncologists and dental professionals.

There are a few limitations with this scoping review. This review may have unintentionally excluded studies due to the lack of clarity on the radiation exposure. Therefore, there could be further reports of dentofacial anomalies following PBT. Additionally, due to the relatively low number of high-energy PBT centres across the world, there is limited data on the late AEs of this treatment in the paediatric literature. The included studies only report the toxicities in patients who have survived and attended follow-up appointments, consequently there may be a higher incidence of dentofacial development disturbances than that reported. As it has not been possible to determine from this review if there is a critical time point after PBT in which dentofacial AEs may be identified, it is sensible to propose that all paediatric cancer survivors are reviewed until their early twenties. This is to allow for complete growth of the facial bones and development of the permament dentition. As with adult head and neck cancer patients, all paediatric patients should receive a pre-treatment dental assessment.^33^, ^34^ This should include radiographic evaluation to allow for the diagnosis of dental diseases. A pre-treatment dental radiograph(s) will also allow a clinician to confirm the absence / presence of a dental developmental disturbance post-treatment. Considering paediatric cancer suvivors are at higher risk of dental caries (decay), dental radiographs, specifically bitewing radiographs are recommended on a 6 monthly basis.^35^ However, the potential limitations of radiographic equipment availability at Oncology centres is recognised. Therefore, if any potential dental-related AEs are suspected, panoramic radiographs could be justified to assess the developing dentition. This will also support the collection of outcome data on dentofacial toxicities. Close liaison and support with dental services is essential in the post-treatment care of paediatric head and neck cancer survivors.

## Conclusion

It can be concluded that radiation to the paediatric facial structures can lead to unintended late consequences with regards to the developing dentition and facial bones. However, considering both the poor study reporting and limited patient numbers, it is not possible to determine the effect of cancer diagnosis, chronological age at treatment, radiation dose or treatment modality on the incidence of facial deformation or dental development anomalies. It may be that it is not possible to minimise these AEs, however if this is the case it is even more important to educate patients on the risks.

The research gap identified by this review can be addressed via more focused research on dentofacial anomalies following PBT, which aims to characterise the dose-effect to the facial bones and teeth, impact of age at treatment and impact of inductive/concurrent chemotherapy. Furthermore, although outside of the scope of this review, it can be argued that there is a need to explore dentofacial outcomes following XRT also. This review also highlighted the need for improved assessments of QoL and patient-reported outcome tools, which would allow for comparisons of dentofacial toxicities across treatment centres.

## CRediT authorship contribution statement

**Emma Foster-Thomas**: Conceptualization, Methodology, Data curation, Writing – original draft. **Marianne Aznar**: Writing – review & editing. **Bernadette Brennan**: Writing − review & editing. **Lucy O’Malley**: Methodology, Supervision, Writing – review & editing.

## Data sharing statement

Not applicable.

## Funding

The first author is undertaking a Doctoral Fellowship, funded by the National Institute for Health and Care Research (NIHR302988). Professor Marianne Aznar acknowledges the support of the 10.13039/501100000266Engineering and Physical Sciences Research Council (Grant number EP/T028017/1).

## Declaration of Competing Interest

The authors declare that they have no known competing financial interests or personal relationships that could have appeared to influence the work reported in this paper.

## References

[1] Cancer Research UK, Children’s cancer statistics. [cited 2020 1 Sep]; Available from: 〈https://www.cancerresearchuk.org/health-professional/cancer-statistics/childrens-cancers〉.

[2] Gan H.W., Spoudeas H.A. (2014). Long-term follow-up of survivors of childhood cancer (SIGN Clinical Guideline 132). Archives of Disease in Childhood Educ Pract Ed.

[3] Schoot R.A. (2017). Facial asymmetry in head and neck rhabdomyosarcoma survivors. Pediatric Blood & Cancer.

[4] Milgrom S. (2020). Dental Abnormalities in Childhood Cancer Survivors Treated with Radiation Therapy to the Head-and-Neck: A Report from the Pediatric Normal Tissue Effects in the Clinic (PENTEC) Group. International Journal of Radiation Oncology, Biology, Physics.

[5] Nemeth O. (2013). Long-term effects of chemotherapy on dental status of children cancer survivors. Pediatric Hematology and Oncology.

[6] NHS England, *Clinical Commissioning Policy: Proton Beam Therapy for Children, Teenagers and Young Adults in the treatment of malignant and non-malignant tumours*. 2020.

[7] Ladra M.M. (2014). A dosimetric comparison of proton and intensity modulated radiation therapy in pediatric rhabdomyosarcoma patients enrolled on a prospective phase II proton study. Radiotherapy and Oncology.

[8] Swanson E.L. (2012). Comparison of three-dimensional (3D) conformal proton radiotherapy (RT), 3D conformal photon RT, and intensity-modulated RT for retroperitoneal and intra-abdominal sarcomas. International Journal of Radiation Oncology** Biology** Physics.

[9] Munn Z. (2018). Systematic review or scoping review? Guidance for authors when choosing between a systematic or scoping review approach. BMC Medical Research Methodology.

[10] Page M.J. (2021). The PRISMA 2020 statement: an updated guideline for reporting systematic reviews. International Journal of Surgery.

[11] Childs S.K. (2012). Proton radiotherapy for parameningeal rhabdomyosarcoma: clinical outcomes and late effects. International Journal of Radiation Oncology - Biology - Physics.

[12] Fitzek M.M., Mukai S., Buzney S.M., Preutt R.C., Adams J., Munzenrider J.E. (2000). Cosmetic Aspects of Proton Irradiation for Retinoblastoma. ABSTRACTS of the XXXII PTCOG MEETING. The Workshop on Computational Methods for Proton Beam Treatment Planning.

[13] Hol M.L. (2020). Dose-Effect Analysis of Early Changes in Orbital Bone Morphology After Radiation Therapy for Rhabdomyosarcoma. Practical Radiation Oncology.

[14] Kharod S.M. (2020). Outcomes following proton therapy for Ewing sarcoma of the cranium and skull base. Pediatric Blood & Cancer.

[15] Ludmir E.B. (2019). Patterns of failure following proton beam therapy for head and neck rhabdomyosarcoma. Radiother Oncol.

[16] Mouw K.W. (2017). Analysis of patient outcomes following proton radiation therapy for retinoblastoma. Advances in radiation oncology.

[17] Fukushima H. (2017). Comorbidity and quality of life in childhood cancer survivors treated with proton beam therapy. Pediatrics International.

[18] Oshiro Y. (2011). Pediatric nasopharyngeal carcinoma treated with proton beam therapy. Two case reports. Acta Oncologica.

[19] Leiser D. (2016). Tumour control and quality of life in children with rhabdomyosarcoma treated with pencil beam scanning proton therapy. Radiotherapy and oncology.

[20] Thompson R.F. (2013). Dose to the developing dentition during therapeutic irradiation: organ at risk determination and clinical implications. International Journal of Radiation Oncology - Biology - Physics.

[21] Hoogeveen R.C. (2020). An overview of radiological manifestations of acquired dental developmental disturbances in paediatric head and neck cancer survivors. Dentomaxillofacial Radiology.

[22] U.S. Department of Health and Human Services. *Common Terminology Criteria for Adverse Events (CTCAE) Version 5.0*. 2017 [cited 2023 January 17]; Available from: 〈https://ctep.cancer.gov/protocoldevelopment/electronic_applications/docs/ctcae_v5_quick_reference_5×7.pdf〉.

[23] Kharod S.M. (2020). Outcomes following proton therapy for Ewing sarcoma of the cranium and skull base. Pediatr Blood Cancer.

[24] Talekar A.L., Musale P.K., Kothare S.S. (2022). Dental Caries and Dental Anomalies in Children Undergoing Chemotherapy for Malignant Diseases. International Journal of Clinical Pediatric Dentistry.

[25] Varni, J.W. *The PedsQL(TM) Measurement Model for the Pediatruc Quality of Life Inventory*. [cited 2021 October]; Available from: 〈http://www.pedsql.org/about_pedsql.html〉. 10.1097/00005650-199902000-00003 10024117

[26] Viswanathan M., A.M, Berkman N.D., et al., *Assessing the Risk of Bias of Individual Studies in Systematic Reviews of Health Care Interventions*. in *In: Methods Guide for Effectiveness and Comparative Effectiveness Reviews [Internet]. Rockville (MD): Agency for Healthcare Research and Quality (US); 2008–. Table A-3, McMaster tool for assessing quality of harms assessment and reporting in study reports (McHarm)* 2012.

[27] Hammond P. (2004). 3D analysis of facial morphology. American Journal of Medical Genetics Part A.

[28] Miller T.P. (2019). Unintended consequences of evolution of the common terminology criteria for adverse events. Pediatric Blood & Cancer.

[29] Bei M. (2009). Molecular genetics of tooth development. Current Opinion in Genetics & Development.

[30] Kaste S.C. (2009). Impact of radiation and chemotherapy on risk of dental abnormalities: a report from the Childhood Cancer Survivor Study. Cancer: Interdisciplinary International Journal of the American Cancer Society.

[31] Kaste S.C. (2009). Impact of radiation and chemotherapy on risk of dental abnormalities: a report from the Childhood Cancer Survivor Study. Cancer.

[32] Timmermann B. (2007). Spot-scanning proton therapy for malignant soft tissue tumors in childhood: First experiences at the Paul Scherrer Institute. International Journal of Radiation Oncology** Biology** Physics.

[33] Royal College of Surgeons. *The Oral Management of Oncology Patients Requiring Radiotherapy, Chemotherapy and / or Bone Marrow Transplantation*. 2018 [cited 2023 January 17]; Available from: 〈https://www.rcseng.ac.uk/dental-faculties/fds/publications-guidelines/clinical-guidelines/〉.

[34] American Academy of Paediatric Dentistry (2016). Guideline on dental management of pediatric patients receiving chemotherapy, hematopoietic cell transplantation, and/or radiation therapy.. Paediatric Dentistry.

[35] Faculty of General Dental Practice (U.K.), *Selection Criteria for Dental Radiography (updated version).* ed. K. Horner, Eaton, KA. 2018.

