# Supplementary material for: Proton beam therapy and dentofacial development in paediatric cancer patients: A scoping review
Source: Int J Part Ther. 2024 Jun 6;12:100107. doi: 10.1016/j.ijpt.2024.100107 (PMC11215291; doi:10.1016/j.ijpt.2024.100107)
Supplement: Supplementary file 1 — Supplementary material [file mmc1.docx]

**Supplementary Material**

***Table 1 - MEDLINE Ovid search strategy.***

| **1** | Adolescent/ or child / or child, preschool/ or infant/ or infant, newborn/ |
| --- | --- |
| **2** | ((head or neck or mouth or oral or lip$1 or tongue or laryn$4 or pharyn$4 or oropharyn$4 or nas$11 or sinus$2 or sinonasal or orbit$2 or brain) adj (cancer$1 or neoplasm$1 or tumo$3 or carcinoma$1 or sarcoma$1)).af |
| **3** | neoplasms/ or skull neoplasms/ or eye neoplasms/ or “head and neck neoplasms”/ |
| **4** | 2 or 3 |
| **5** | 1 and 4 |
| **6** | Proton Therapy/ |
| **7** | particle therapy.af |
| **8** | cancer therapy/ |
| **9** | or/6-8 |
| **10** | Facial Asymmetry/ |
| **11** | dentofacial development.af |
| **12** | mouth abnormalities/or hypercementosis/ or malocclusion/ or tooth abnormalities/ or tooth ankylosis/ or tooth eruption, ectopic/ or tooth loss/ or tooth resorption/ or tooth, impacted/or tooth, unerupted/ |
| **13** | Maxillofacial Development/ |
| **14** | or/10-13 |
| **15** | 5 and 9 and 14 |

***Table 2: Cancer diagnoses reported in the included studies***

|  | **Cancer diagnosis** | **Dentofacial anomalies reported** |
| --- | --- | --- |
| Childs 2012 | Parameningeal Rhabdomyosarcoma | Mild facial hypoplasia (n=7)  Failure of permanent tooth eruption adjacent to the treatment field (n=3) |
| Fitzek 2000 | Retinoblastoma | Facial asymmetry (n=3) |
| Fukushima 2017 | Ewing sarcoma – Grade 1 and Grade 3  Rhabdomyosarcoma – Grade 3  Brain tumour (n=10)  RMS (n=8)  Ewing (n=4)  Nasopharyngeal (n=3)  Chordoma (n=2)  Neuroblastoma (n=2)  Other (n=3) | Facial deformity (n=8)  Dental impairment (n=3) |
| Hol 2020 | Embryonal RMS | Orbital volume changes (16/17) |
| Hoogeveen 2020 | Spindle cell rhabdomyosarcoma right nasopharynx | Root stunting, arrest of root formation, failed tooth eruption |
| Kharod 2019 | Ewing sarcoma | Delay in tooth eruption (1/25) |
| Leiser 2016 | Rhabdomyosarcoma | Facial hypoplasia (14/83)  Parameningeal RMS (9/46)  Orbital RMS (5/17)  Dental impairment (3/46)  Parameningeal RMS |
| Ludmir 2019 | Rhabdomyosarcoma | Bone hypoplasia and facial asymmetry (n=2) – does not specify which bone or given further details of site of tumour |
| Mouw 2017 | Retinoblastoma | Mid facial asymmetry, specifically maxilla (1/12) |
| Oshiro 2011 | Nasopharyngeal carcinoma | Retardation of mandibular ramus in treatment field, facial asymmetry not visible  Atrophy of lower right molars (however looks like extensive caries) |
| Thompson 2013 | Rhabdomyosarcoma (n=10)  Sarcoma (n=2)  Teratoma (n=1)  Parotid carcinoma (n=1) | Abnormal or missing teeth (n=2) – one had retrobulbar teratoma  Asymmetrically delayed tooth eruption (n=1) – orbital rhabdomyosarcoma |
